# Sociodemographic, Lifestyle, and Quality of Life Determinants of Atherogenic Risk: A Cross-Sectional Study in a Large Cohort of Spanish Workers

**DOI:** 10.3390/jcm14196876

**Published:** 2025-09-28

**Authors:** María Dolores Marzoa Jansana, Pedro Juan Tárraga López, Juan José Guarro Miquel, Ángel Arturo López-González, Pere Riutord Sbert, Carla Busquets-Cortés, José Ignacio Ramírez-Manent

**Affiliations:** 1 ADEMA-Health Group, University Institute for Research in Health Sciences (IUNICS), 07010 Palma, Spainc.busquets@eua.edu.es (C.B.-C.);; 2 Faculty of Medicine, UCLM (University of Castilla La Mancha), 02008 Albacete, Spain; 3 Faculty of Dentistry, ADEMA—Universidad de las Islas Baleares, 07010 Palma, Spain; 4 Balearic Islands Health Research Institute Foundation (IDISBA), 07010 Palma, Spain; 5 Balearic Islands Health Service, 07010 Palma, Spain; 6 Faculty of Medicine, University of the Balearic Islands, 07010 Palma, Spain

**Keywords:** atherogenic risk, atherogenic dyslipidemia, quality of life, life style, mediterranean diet, socioeconomic variables

## Abstract

**Background:** Atherosclerosis is a leading cause of cardiovascular morbidity and mortality worldwide. Although lipid-derived atherogenic indices are widely used for cardiovascular risk assessment, their relationship with sociodemographic factors, lifestyle behaviors, and health-related quality of life (HRQoL) in occupational populations remains insufficiently explored. This study aimed to evaluate the association between atherogenic risk, measured by total cholesterol/high-density lipoprotein cholesterol (TC/HDL-c), low-density lipoprotein cholesterol/high-density lipoprotein cholesterol (LDL-c/HDL-c), triglyceride/high-density lipoprotein cholesterol (TG/HDL-c), and atherogenic dyslipidemia (AD) and sociodemographic, lifestyle, and HRQoL variables in a large cohort of Spanish workers. **Methods:** We conducted a cross-sectional analysis of 100,014 Spanish workers aged 18–69 years, of whom 39.9% were women, with a mean age of 38.2 years (SD 10.2 or IQR) and 38.9 years (SD 10.3 or IQR) for men, during the health examinations carried out between 2021 and 2024. Sociodemographic variables included sex, age group, and occupational social class. Lifestyle factors comprised smoking status, adherence to the Mediterranean diet (MEDAS score), and physical activity (IPAQ categories). HRQoL was assessed using the 12-item Short Form Survey (SF-12), stratified into good vs. poor categories. Logistic regression models were applied to estimate odds ratios (OR) and 95% confidence intervals (CI) for moderate-to-high atherogenic risk across indices, adjusting for potential confounders. **Results:** Men exhibited a lower likelihood of moderate-to-high TC/HDL-c and LDL-c/HDL-c but a markedly higher probability of elevated TG/HDL-c and AD compared to women (OR range: 0.42–3.67, *p* < 0.001). A clear age-related gradient was observed across all indices, with participants aged 60–69 showing the highest risk (OR range: 2.28–7.84, *p* < 0.001). Lower social class, smoking, physical inactivity, poor diet, and poor SF-12 scores were significantly associated with increased atherogenic risk, with physical inactivity (OR up to 8.61) and poor diet (OR up to 4.98) emerging as the strongest predictors. **Conclusions:** Atherogenic risk in this large working cohort is strongly influenced by both traditional cardiovascular risk factors and HRQoL. Integrating lifestyle modification and quality-of-life improvement strategies into workplace health programs could substantially reduce the atherogenic burden. Longitudinal research is needed to confirm these associations and guide targeted interventions.

## 1. Introduction

Atherosclerosis is the leading cause of cardiovascular morbidity and mortality worldwide [1,2]. Imaging and autopsy studies have demonstrated that a large proportion of older adults, particularly those over 60 years of age, present with subclinical atheromatous plaques [3,4]. The disease process is initiated by endothelial dysfunction, facilitating the retention and oxidative modification of low-density lipoprotein cholesterol (LDL-c) within the arterial intima, followed by monocyte recruitment, foam cell formation, and sustained arterial inflammation [5,6]. Reactive oxygen species (ROS) play a pivotal role in promoting lipid oxidation, impairing endothelial nitric oxide production, and weakening the fibrous cap covering plaques [7,8].

High-density lipoprotein cholesterol (HDL-c) exerts multiple protective effects, including reverse cholesterol transport, inhibition of LDL oxidation, reduction of oxidative stress, maintenance of endothelial nitric oxide synthase (eNOS) activity, prevention of endothelial apoptosis, and anti-inflammatory modulation of the vascular wall [9,10,11]. However, HDL functionality is heterogeneous and can be compromised in certain metabolic conditions despite normal HDL-C concentrations [12].

In clinical practice, risk stratification tools such as the Framingham Risk Score and SCORE combine demographic, clinical, and lipid data to estimate the probability of future cardiovascular events [13,14]. Nevertheless, residual cardiovascular risk often persists even when LDL-c targets are achieved [15]. In this context, lipid ratios—including total cholesterol to HDL-c (TC/HDL-c), LDL-c to HDL-c (LDL-c/HDL-c), and triglycerides to HDL-c (TG/HDL-c)—have emerged as valuable prognostic markers [16,17]. These composite indices reflect both pro-atherogenic and anti-atherogenic components, offering a more comprehensive assessment of cardiovascular risk than single lipid measures [18]. Elevated TG/HDL-c ratios, in particular, are strongly associated with increased incidence of major adverse cardiovascular events (MACE; i.e., myocardial infarction, stroke, cardiovascular death, and heart failure), cardiovascular mortality, and plaque progression, independently of traditional risk factors and LDL-c levels [19,20,21]. Studies in patients with acute coronary syndrome undergoing percutaneous coronary intervention have shown that higher baseline TG/HDL-c predicts greater long-term MACE risk [22], while elevated LDL-c/HDL-c ratios correlate with greater severity of coronary artery disease and higher plaque burden [23,24].

Quality of life (QoL) is increasingly recognized as a critical determinant of cardiometabolic health, encompassing subjective perceptions of physical, psychological, and social well-being rather than the mere absence of disease [25]. In working populations, better QoL scores are associated with healthier behaviors, greater adherence to preventive measures, and improved long-term metabolic outcomes [26,27]. The 12-Item Short Form Survey (SF-12) is a widely used and validated instrument for large-scale studies, capturing both physical and mental health domains with high reliability and minimal respondent burden [28]. Lower SF-12 scores have been linked to higher prevalence of metabolic syndrome, increased incidence of cardiovascular events, and greater healthcare utilization [29,30,31].

The relationship between QoL and cardiometabolic risk is likely bidirectional. On one hand, individuals with metabolic disturbances or subclinical atherosclerosis may experience reduced functional capacity, fatigue, and psychological distress, leading to lower QoL scores [32,33]. On the other hand, poor QoL may act as a chronic psychosocial stressor, promoting unhealthy behaviors, disrupting neuroendocrine regulation, and enhancing inflammatory pathways that accelerate vascular injury and dyslipidemia [34,35].

Insulin resistance is closely linked to vascular dysfunction and dyslipidemia and contributes to the progression of atherosclerosis and cardiovascular events [36].

Despite extensive evidence on individual lifestyle factors and lipid abnormalities, little is known about their joint associations with atherogenic indices in occupational settings. Spain, with its structured occupational health surveillance system and heterogeneous workforce, offers an ideal context to examine these relationships [37]. Therefore, the aim of this study was to investigate the relationship between sociodemographic, lifestyle, and health-related quality of life variables with atherogenic lipid indices in a large cohort of Spanish workers. We hypothesized that adverse sociodemographic conditions, unhealthy lifestyle behaviors, and lower HRQoL would be independently associated with higher atherogenic risk.

## 2. Materials and Methods

### 2.1. Study Design and Population

**Figure 1:**
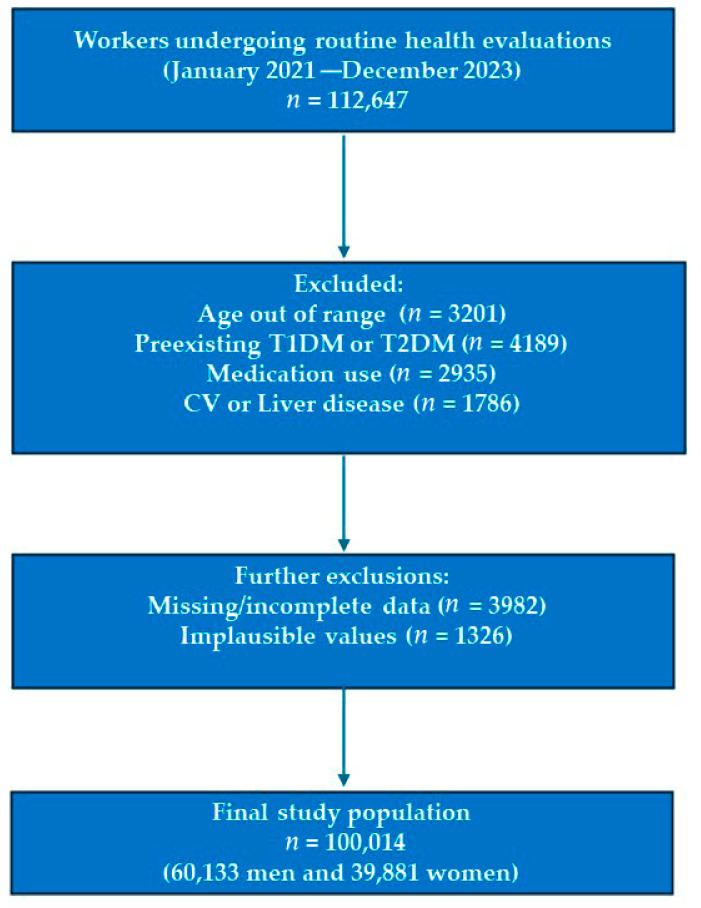
Flow Chart: Participant Selection Process.

A cross-sectional analysis was conducted within the framework of a nationwide occupational health surveillance program in Spain. The study population comprised 100,014 actively employed individuals (60,133 men and 39,881 women) aged 18–69 years, evaluated between January 2021 and December 2023 during routine workplace health assessments (Figure 1). Participants were recruited through certified occupational health centers distributed across Spain. These centers were selected based on their accreditation status and geographical distribution to ensure national representativeness.

Given the availability of a large occupational cohort evaluated during routine health check-ups, we chose a cross-sectional design. This design allows for the identification of associations between atherogenic indices and sociodemographic, lifestyle, and health-related quality of life variables. However, causal relationships cannot be established. The study should therefore be considered hypothesis-generating and a foundation for future longitudinal research.

The study adhered to the STROBE guidelines for cross-sectional studies, and the completed STROBE checklist is provided as Supplementary Material.

### 2.2. Anthropometric and Clinical Measurements

All measurements were obtained by trained health professionals using standardized procedures. Height and weight were recorded with participants in light clothing and without shoes. Body mass index (BMI) was calculated as weight (kg) divided by squared height (m^2^). Waist circumference was measured at the midpoint between the lower rib margin and the iliac crest. Blood pressure was recorded in triplicate after five minutes of seated rest using a validated automated sphygmomanometer.

Venous blood samples were collected after at least eight hours of fasting to determine glucose, total cholesterol, HDL-cholesterol, LDL-cholesterol, and triglyceride concentrations. Analyses were performed in accredited laboratories using enzymatic colorimetric methods and following internal and external quality control protocols.

### 2.3. Atherogenic Risk Assessment

Atherogenic dyslipidemia was defined as the coexistence of elevated triglycerides (≥150 mg/dL) and low HDL-c (<40 mg/dL in men and <50 mg/dL in women), in accordance with international criteria. This operational definition has been widely used in epidemiological and occupational health studies to capture the combined lipid abnormalities associated with increased cardiometabolic risk.

The cut-off values for the main atherogenic indices vary depending on the parameter evaluated. For the total cholesterol/HDL-c ratio, low risk is defined as <5 in men and <4.5 in women; moderate risk corresponds to values between 5 and 9 in men and between 4.5 and 7 in women; and high risk is indicated by >9 in men and >7 in women [38]. For the LDL-c/HDL-c ratio, low risk is <3, whereas values ≥ 3 indicate high risk [39]. For the triglycerides/HDL-c ratio, a value ≥ 3 is generally considered indicative of high cardiovascular risk [40].

In addition, the presence of atherogenic dyslipidemia was defined as the combination of elevated triglycerides and reduced HDL-c, according to international consensus criteria [41,42].

### 2.4. Lifestyle and Behavioral Variables

Adherence to the Mediterranean diet was assessed with the validated 14-item Mediterranean Diet Adherence Screener (MEDAS) from the PREDIMED study; scores ≥ 9 denoted adequate adherence [43,44]. Physical activity was evaluated using the International Physical Activity Questionnaire–Short Form (IPAQ-SF), categorizing participants as active or inactive based on MET-min/week thresholds from international guidelines [45]. The MEDAS questionnaire assesses adherence to the Mediterranean diet based on 14 items related to food intake patterns, while the short-form IPAQ evaluates physical activity across different domains of daily life, expressed in MET-minutes/week. Smoking status was self-reported as current smoker or non-smoker.

### 2.5. Sociodemographic Variables

Data were collected on sex, age, and occupational social class, classified according to the 2011 Spanish National Classification of Occupations (CNO-11) and the recommendations of the Spanish Society of Epidemiology into [46]:

Occupational social class was categorized as: Class I (managers and professionals, e.g., directors, engineers, physicians), Class II (intermediate occupations, e.g., clerical and technical staff), and Class III (manual and unskilled workers, e.g., factory workers, cleaners, construction laborers).

### 2.6. Health-Related Quality of Life

Quality of life was measured using the 12-Item Short Form Health Survey (SF-12), generating two composite scores: the Physical Component Summary (PCS) and the Mental Component Summary (MCS) [47]. Based on median values for the study population, participants were categorized as having “good” or “poor” health-related quality of life (HRQoL).

### 2.7. Statistical Analysis

Continuous variables were expressed as mean ± standard deviation, and categorical variables as absolute and relative frequencies. Group differences were evaluated using Student’s *t*-test or ANOVA for continuous variables, and the chi-square test for categorical variables. Trends across ordered categories (e.g., age, social class) were examined using linear regression or Cochran–Armitage tests. For trend analyses, age was included as a continuous variable, whereas social class and lifestyle categories were analyzed as ordered categories.

Normality of continuous variables was assessed using Kolmogorov–Smirnov tests and visual inspection. Although some deviations were observed due to the large sample size, parametric models were used as they are robust to minor departures from normality and allowed comparability across subgroups.

To assess the association between sociodemographic, lifestyle, and HRQoL variables with each atherogenic risk indicator, multivariate logistic regression models were con-structed, adjusting for relevant confounders. Multivariable logistic regression models were adjusted for age, sex, occupational social class, smoking, diet, physical activity, and HRQoL. Variance inflation factors (VIF) were examined, with no evidence of collinearity detected. Odds ratios (OR) with 95% confidence intervals (CI) were reported.

In addition to categorical classifications, atherogenic indices were analyzed as continuous variables using multivariable linear regression models adjusted for sociodemographic and lifestyle covariates.

Missing data were examined for all variables. The proportion of missing data was <5% for sociodemographic and lifestyle factors and <7% for HRQoL variables. Individuals with incomplete data for the main outcomes were excluded from multivariable analyses. Sensitivity analyses using multiple imputation yielded similar results, supporting the robustness of the findings.

All analyses were conducted using IBM SPSS Statistics version 29.0 (IBM Corp., Armonk, NY, USA). Statistical significance was set at *p* < 0.05.

## 3. Results

Table 1 summarizes the baseline characteristics of 100,014 workers stratified by sex. Marked sex-related differences are evident: men exhibit higher mean values for weight, waist circumference, blood pressure, triglycerides, and glucose, whereas women show higher HDL-c levels. Differences are also observed in age distribution, occupational class, smoking, Mediterranean diet adherence, and physical activity, with women reporting healthier lifestyle patterns overall. These findings highlight the need for sex-stratified analyses and adjustment in multivariate models, as sex strongly influences cardiometabolic risk profiles.

Table 2 displays the distribution of mean lipid ratios across population subgroups. A consistent age gradient is observed, with higher ratios in older participants of both sexes. Lower occupational class, smoking, poor Mediterranean diet adherence, physical inactivity, and low SF-12 scores are associated with less favorable lipid profiles. The magnitude of the differences between physically active versus inactive participants and between those adhering or not adhering to the Mediterranean diet highlights the crucial role of lifestyle behaviors in modulating atherogenic risk.

Table 3 presents age-standardized prevalence rates of moderate-to-high TC/HDL-c, LDL-c/HDL-c, TG/HDL-c, and atherogenic dyslipidemia. The results confirm a strong age-related gradient and higher prevalence among lower social classes and smokers. Adherence to the Mediterranean diet and engagement in physical activity markedly reduce prevalence, whereas poor SF-12 scores are associated with substantial increases across all indices. These patterns demonstrate the additive and synergistic impact of lifestyle factors and perceived health status on cardiometabolic risk.

When analyzed as continuous variables, higher triglyceride-to-HDL-c and LDL-c-to-HDL-c ratios were consistently associated with lower social class, smoking, physical inactivity, and poorer HRQoL. These associations paralleled those obtained with categorical classifications.

Table 4 reports adjusted odds ratios for the associations between sociodemographic, behavioral, and quality-of-life factors with the four atherogenic risk outcomes. Male sex is inversely associated with elevated TC/HDL-c and LDL-c/HDL-c but strongly and positively associated with high TG/HDL-c and atherogenic dyslipidemia. Advancing age, manual occupational class, smoking, poor diet, and physical inactivity all demonstrate robust associations with adverse lipid outcomes. Notably, physical inactivity (OR up to 8.61) and poor diet (OR up to 4.98) emerge as the strongest predictors. Poor SF-12 scores further amplify risk, emphasizing the relevance of integrating patient-reported quality-of-life measures into cardiometabolic risk assessment.

**Figure 2:**
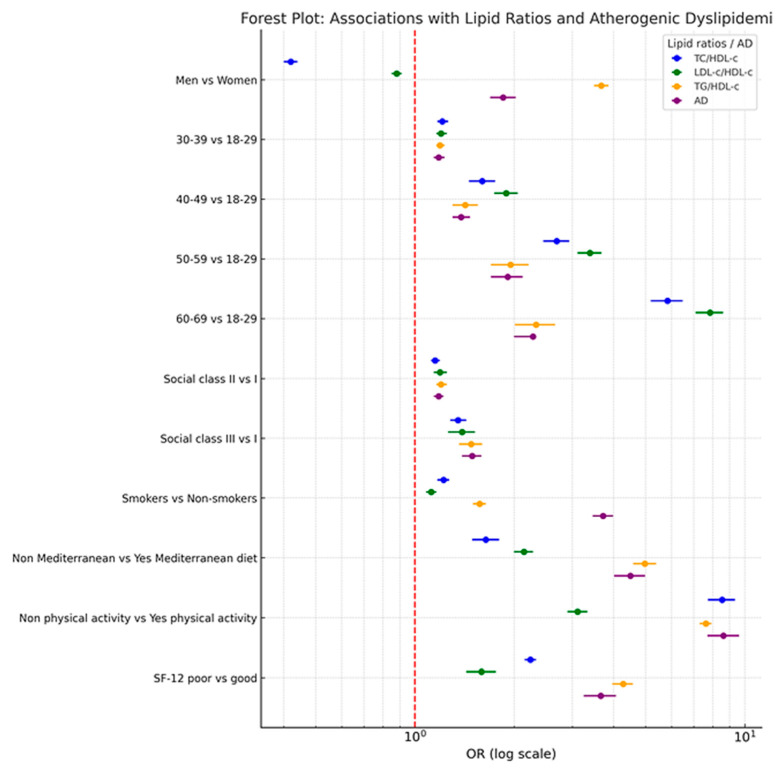
Forest Plot: Associations with Lipid Ratios and Atherogenic Dyslipidemia.

Figure 2 Forest Plot of Multivariate Associations Between Sociodemographic, Lifestyle, and Quality-of-Life Variables and Atherogenic Risk Outcomes.

Figure 2 visually summarizes adjusted odds ratios (ORs) and 95% confidence intervals for the four lipid-derived risk markers. The forest plot highlights protective associations (OR < 1) and risk-enhancing associations (OR > 1) across subgroups. Key findings include an inverse association of male sex with TC/HDL-c and LDL-c/HDL-c and a positive association with TG/HDL-c and atherogenic dyslipidemia, as well as pronounced risk elevations linked to physical inactivity and poor diet. The logarithmic scale and color-coded representation facilitate interpretation of both the magnitude and consistency of associations across outcomes, providing a clear synthesis of complex multivariable results.

Sensitivity analyses stratified by sex and age groups yielded results consistent with the main models. Excluding individuals with extreme values of lipid indices or with self-reported lifestyle variables did not materially alter the associations, confirming the robustness of our findings.

## 4. Discussion

This large cross-sectional study identified significant associations between sociodemographic factors, health behaviors, and quality of life with multiple atherogenic risk indices, including TC/HDL-c, LDL-c/HDL-c, TG/HDL-c, and atherogenic dyslipidemia (AD). Men showed markedly higher odds for TG/HDL-c and AD, while women had higher odds for TC/HDL-c and LDL-c/HDL-c in the moderate-to-high range. Increasing age was consistently associated with elevated risk across all indices, with the highest odds in participants aged 60–69 years. Lower social class, smoking, low adherence to the Mediterranean diet, physical inactivity, and poor SF-12 scores were robustly associated with higher atherogenic risk, highlighting the cumulative effect of adverse lifestyle factors and poorer perceived health status on lipid-related cardiovascular risk.

Our findings are consistent with previous population-based and occupational studies that demonstrate the predictive value of atherogenic indices for cardiovascular morbidity and mortality [48,49]. In particular, TC/HDL-c and LDL-c/HDL-c ratios have been consistently associated with coronary artery disease risk in both general and working populations [50,51]. Similarly, TG/HDL-c has been recognized as a surrogate marker for insulin resistance and metabolic syndrome, strongly linked to atherosclerosis progression [52,53,54].

The strong relationship between poor adherence to the Mediterranean diet and elevated atherogenic indices in our cohort reflects previous evidence demonstrating its protective role against dyslipidemia, systemic inflammation, and endothelial dysfunction [55,56,57]. Likewise, physical inactivity, one of the strongest determinants in our analysis, has been shown to contribute to atherogenic lipid profiles and higher carotid intima–media thickness [58,59,60,61].

An important finding of our study is the independent association between health-related quality of life (HRQoL), as measured by the SF-12 scores, and atherogenic risk. This association suggests that psychosocial and subjective health dimensions may play a role in lipid dysregulation. Several mechanisms may underlie this relationship, including the impact of stress and poor mental well-being on inflammatory pathways, neuroendocrine function, and adoption of unhealthy behaviors. Previous studies have also reported links between lower HRQoL and adverse cardiometabolic outcomes, supporting our results. Integrating HRQoL assessment into occupational health evaluations may therefore provide a broader understanding of cardiovascular risk beyond traditional clinical and lifestyle factors.

Regarding quality of life, although few studies have directly linked SF-12 scores with atherogenic indices, evidence supports an inverse association between physical and mental health status and cardiovascular outcomes [62,63]. Poor self-reported health has been associated with higher rates of metabolic syndrome [64], type 2 diabetes [65], and cardiovascular disease [66], likely mediated through behavioral, psychosocial, and physiological pathways. In occupational settings, lower quality-of-life scores have been linked to higher cardiometabolic risk, including hypertension, central obesity, and hypertriglyceridemia [67,68,69,70,71].

This suggests that quality of life is not only a consequence of chronic disease but may also serve as a predictor or early marker of adverse cardiometabolic profiles, reinforcing the relevance of integrating patient-reported outcomes into cardiovascular risk assessments.

This study provides novel evidence by integrating four validated atherogenic indices with sociodemographic, behavioral, and quality-of-life variables in a large and diverse Spanish working population. To our knowledge, it is one of the first to evaluate SF-12 scores alongside lipid-derived risk ratios in such a large occupational cohort, offering a comprehensive view of both biological and self-reported determinants of cardiovascular risk.

Our findings suggest that investigating how sociodemographic, lifestyle, and perceived health factors jointly influence atherogenic lipid ratios may help design workplace-based preventive strategies to mitigate cardiovascular risk and promote long-term health.

The results highlight the potential for incorporating atherogenic indices and quality-of-life assessments into routine occupational health screenings. Future longitudinal research should determine causal relationships and explore whether interventions aimed at improving diet, physical activity, and mental well-being translate into measurable improvements in lipid ratios and reduction in cardiovascular events. Interdisciplinary strategies that combine medical, nutritional, and psychological support may be particularly effective.

### Strengths and Limitations

Strengths of this study include its large sample size, diversity across sectors, and use of validated indices and questionnaires. The simultaneous evaluation of four atherogenic indices and the integration of SF-12 scores provide a multifaceted understanding of cardiovascular risk in working adults. Furthermore, the standardized data collection and adjustment for multiple confounders enhance internal validity. The consistency of results across sensitivity analyses further supports the validity of our conclusions.

However, limitations must be acknowledged. A major limitation of the study is its cross-sectional design, which precludes causal inference. Longitudinal follow-up would be necessary to confirm whether the observed associations translate into incident cardiometabolic events. Variables such as smoking status, dietary adherence, physical activity, and SF-12 scores were self-reported, which may introduce recall or reporting bias, as well as social desirability bias, potentially leading to an underestimation of prevalence estimates. Some relevant biochemical markers, such as apolipoprotein B or inflammatory biomarkers, were not available. Additionally, while the cohort was large, it consisted of employed individuals, which may limit generalizability to unemployed or retired populations.

## 5. Conclusions

This large cross-sectional study demonstrates that atherogenic risk, measured through multiple lipid-derived indices, is strongly associated with sociodemographic characteristics, health behaviors, and quality of life in a diverse Spanish working population. Older age, male sex, lower socioeconomic status, smoking, physical inactivity, poor adherence to the Mediterranean diet, and lower SF-12 physical and mental health scores were consistently linked to elevated atherogenic profiles.

The integration of validated atherogenic indices with patient-reported quality-of-life measures offers a comprehensive approach to cardiovascular risk assessment in occupational health settings. Our findings suggest that preventive strategies should not only target traditional biological risk factors but also address modifiable lifestyle behaviors and psychosocial determinants of health.

Future research should prioritize longitudinal designs to clarify causal relationships and assess the effectiveness of workplace-based interventions integrating nutritional, physical activity, and mental health programs in reducing atherogenic burden and improving overall quality of life.

## Figures and Tables

**Table 1:** Baseline Sociodemographic, Anthropometric, and Clinical Characteristics of the Study Population by Sex and in Total.

Variable	Total (*n* = 100,014)	Men (*n* = 60,133)	Women (*n* = 39,881)	*p*-Value
Age, years (mean ± SD)	44.8 ± 9.6	45.2 ± 9.5	44.1 ± 9.7	<0.001
BMI, kg/m^2^ (mean ± SD)	26.7 ± 4.3	27.3 ± 4.1	25.9 ± 4.4	<0.001
Waist circumference, cm	89.6 ± 12.7	94.1 ± 11.4	82.5 ± 11.1	<0.001
Systolic BP, mmHg	123.4 ± 14.9	126.1 ± 14.7	119.1 ± 14.2	<0.001
Diastolic BP, mmHg	77.6 ± 9.8	79.2 ± 9.6	75.1 ± 9.4	<0.001
Glucose, mg/dL	96.3 ± 16.5	98.2 ± 16.7	93.7 ± 16.0	<0.001
Total cholesterol, mg/dL	199.1 ± 36.2	198.6 ± 35.9	199.8 ± 36.6	0.02
HDL-c, mg/dL	55.8 ± 14.7	50.1 ± 12.5	64.2 ± 14.2	<0.001
Triglycerides, mg/dL	123.7 ± 85.4	136.8 ± 91.3	105.4 ± 72.1	<0.001
Current smokers, %	25.8	28.5	21.4	<0.001
Physical activity (≥150 min/week)	42.6	39.3	47.5	<0.001
Mediterranean diet adherence, %	36.2	33.1	40.8	<0.001

BP, Blood pressure. HDL, High density lipoprotein. LDL, Low density lipoprotein. SD, Standard deviation. Notes: Values are presented as mean ± standard deviation or percentage, as appropriate. Comparisons between sexes were performed using Student’s *t*-test (continuous variables) or chi-squared test (categorical variables).

**Table 2:** Mean Values of Atherogenic Lipid Ratios (TC/HDL-c, LDL-c/HDL-c, TG/HDL-c) Across Sociodemographic, Lifestyle, and Quality-of-Life Subgroups.

		TC/HDL-c		LDL-c/HDL-c		TG/HDL-c	
Men	*n*	Mean (SD)	*p*-Value	Mean (SD)	*p*-Value	Mean (SD)	*p*-Value
18–29 years	10,774	3.2 (0.8)	<0.001	1.8 (0.7)	<0.001	1.8 (1.4)	<0.001
30–39 years	19,795	3.7 (1.0)		2.3 (0.8)		2.4 (2.1)	
40–49 years	17,850	4.2 (1.2)		2.7 (1.0)		2.9 (2.5)	
50–59 years	9915	4.5 (1.2)		2.9 (1.0)		3.0 (1.9)	
60–69 years	1799	4.6 (1.2)		3.0 (1.0)		3.2 (2.5)	
Social class I	3208	3.9 (1.1)	<0.001	2.4 (0.9)	<0.001	2.4 (1.9)	<0.001
Social class II	10,602	4.0 (1.1)		2.5 (0.9)		2.5 (2.2)	
Social class III	46,323	4.0 (1.2)		2.5 (1.0)		2.6 (2.3)	
Smokers	22,265	3.9 (1.1)	<0.001	2.4 (0.9)	<0.001	2.4 (1.8)	<0.001
Non-smokers	37,868	4.0 (1.3)		2.5 (1.1)		2.9 (2.8)	
Adherence to the Mediterranean diet	24,790	3.3 0.7)	<0.001	2.0 (0.6)	<0.001	1.6 (0.7)	<0.001
Non-adherence to the Mediterranean diet	35,343	4.4 (1.2)		2.7 (1.1)		3.3 (2.6)	
Perform physical activity	27,551	3.4 (0.7)	<0.001	2.1 (0.7)	<0.001	1.6 (0.6)	<0.001
Physical inactivity	32,582	4.4 (1.3)		2.8 (1.1)		3.4 (2.7)	
SF-12 normal	41,843	3.7 (0.9)	<0.001	2.3 (0.8)	<0.001	1.8 (0.9)	<0.001
SF-12 low	18,290	4.6 (1.4)		2.8 (1.2)		4.2 (3.3)	
**Women**	***n***	**Mean (SD)**	***p*-Value**	**Mean (SD)**	***p*-Value**	**Mean (SD)**	***p*-Value**
18–29 years	7747	3.2 (0.8)	<0.001	1.9 (0.7)	<0.001	1.4 (0.8)	<0.001
30–39 years	13,365	3.5 (1.0)		2.2 (0.9)		1.5 (0.9)	
40–49 years	11,626	3.9 (1.0)		2.5 (0.9)		1.8 (1.1)	
50–59 years	6121	4.4 (1.1)		3.0 (1.0)		2.1 (1.3)	
60–69 years	1022	4.5 (1.0)		3.1 (0.9)		2.2 (1.0)	
Social class I	2793	3.6 (1.0)	<0.001	2.2 (0.9)	<0.001	1.5 (0.8)	<0.001
Social class II	13,255	3.7 (1.0)		2.3 (0.9)		1.6 (1.0)	
Social class III	23,833	3.8 (1.1)		2.4 (1.0)		1.7 (1.0)	
Smokers	13,040	3.7 (1.1)	<0.001	2.4 (1.0)	<0.001	1.8 (1.1)	<0.001
Non-Smokers	26,841	3–6 (1.0)		2.3 (0.9)		1.7 (1.0)	
Adherence to the Mediterranean diet	20,344	3.3 (0.7)	<0.001	2.0 (0.7)	<0.001	1.3 (0.5)	<0.001
Non-adherence to the Mediterranean diet	19,537	4.2 (1.1)		2.8 (1.1)		2.1 (1.2)	
Perform physical activity	20,669	3.2 (0.7)	<0.001	2.0 (0.6)	<0.001	1.3 (0.4)	<0.001
Physical inactivity	19,212	4.2 (1.1)		2.8 (1.1)		2.2 (1.2)	
SF-12 normal	32,173	3.5 (0.9)	<0.001	2.2 (0.9)	<0.001	1.5 (0.6)	<0.001
SF-12 low	7708	4.6 (1.1)		3.1 (1.1)		2.7 (1.6)	

TC, Total cholesterol. HDL, High density lipoprotein. LDL, Low density lipoprotein. TG, Triglycerides. SF-12, Short Form Health Survey. SD, Standard deviation.

**Table 3:** Age-Standardized Prevalence of Moderate-to-High Atherogenic Lipid Ratios and Atherogenic Dyslipidemia by Population Subgroups.

		TC/HDL-c Moderate-High		LDL-c/HDL-c High		TG/HDL-c High		AD	
Men	*n*	%	*p*-Value	%	*p*-Value	%	*p*-Value	%	*p*-Value
18–29 years	10,774	2.6	<0.001	5.3	<0.001	9.9	<0.001	1.7	<0.001
30–39 years	19,795	8.7		15.9		19.8		4.0	
40–49 years	17,850	18.6		30.6		30.4		6.1	
50–59 years	9915	29.0		43.2		34.7		8.1	
60–69 years	1799	29.2		47.0		36.3		8.4	
Social class I	3208	13.6	<0.001	22.8	<0.001	20.8	<0.001	3.7	<0.001
Social class II	10,602	14.5		23.9		24.3		4.5	
Social class III	46,323	14.8		25.7		24.6		4.9	
Smokers	22,265	15.9	<0.001	24.2	<0.001	27.9	<0.001	9.4	<0.001
Non-smokers	37,868	13.7		23.5		22.2		2.1	
Adherence to MD	24,790	8.3	<0.001	13.9	<0.001	14.5	<0.001	3.7	<0.001
Non-adherence to MD	35,343	21.9		31.7		34.0		8.5	
Perform PhA	27,551	7.0	<0.001	10.2	<0.001	12.8	<0.001	3.0	<0.001
Physical inactivity	32,582	23.8		37.9		38.0		9.7	
SF-12 normal	41,843	7.6	<0.001	18.6	<0.001	8.7	<0.001	4.2	<0.001
SF-12 low	18,290	30.2		35.7		60.3		9.9	
**Women**	***n***	**%**	***p*-Value**	**%**	***p*-Value**	**%**	***p*-Value**	**%**	***p*-Value**
18–29 years	7747	6.2	<0.001	7.3	<0.001	4.8	<0.001	0.8	<0.001
30–39 years	13,365	13.6		15.5		5.7		1.4	
40–49 years	11,626	24.0		26.3		8.9		2.6	
50–59 years	6121	43.5		46.4		15.8		6.0	
60–69 years	1022	46.8		50.2		17.8		6.1	
Social class I	2793	16.5	<0.001	19.5	<0.001	5.1	<0.001	1.9	<0.001
Social class II	13,255	18.8		20.9		7.7		2.2	
Social class III	23,833	22.1		24.1		9.0		2.7	
Smokers	13,040	21.6	<0.001	23.9	<0.001	8.9	<0.001	2.5	<0.001
Non-Smokers	26,841	18.6		20.3		8.0		2.4	
Adherence to MD	20,344	14.9	<0.001	14.8	<0.001	5.9	<0.001	1.2	<0.001
Non-adherence to MD	19,537	27.9		30.2		10.9		4.2	
Perform PhA	20,669	10.1	<0.001	12.5	<0.001	5.0	<0.001	0.9	<0.001
Physical inactivity	19,212	30.9		33.6		12.0		4.8	
SF-12 normal	32,173	13.2	<0.001	16.0	<0.001	6.9	<0.001	1.4	<0.001
SF-12 low	7708	51.5		50.5		19.9		5.6	

TC, Total cholesterol. HDL, High density lipoprotein. LDL, Low density lipoprotein. TG, Triglycerides. AD, Atherogenic dyslipidemia. MD, Mediterranean diet. PhA, Physical activity. SF-12, Short Form Health Survey.

**Table 4:** Multivariate Logistic Regression of Sociodemographic, Lifestyle, and Quality-of-Life Determinants of Atherogenic Risk.

	TC/HDL-c Moderate-High		LDL-c/HDL-c High		TG/HDL-c High		AD	
	OR (95% CI)	*p*-Value	OR (95% CI)	*p*-Value	OR (95% CI)	*p*-Value	OR (95% CI)	*p*-Value
Women	1		1		1		1	
Men	0.42 (0.40–0.44)	<0.001	0.88 (0.85–0.91)	<0.001	3.67 (3.49–3.86)	<0.001	1.85 (1.69–2.02)	<0.001
18–29 years	1		1		1		1	
30–39 years	1.21 (1.17–1.26)	<0.001	1.20 (1.16–1.25)	<0.001	1.19 (1.16–1.23)	<0.001	1.18 (1.14–1.23)	<0.001
40–49 years	1.60 (1.46–1.75)	<0.001	1.89 (1.74–2.05)	<0.001	1.42 (1.30–1.55)	<0.001	1.38 (1.30–1.47)	<0.001
50–59 years	2.69 (2.45–2.94)	<0.001	3.39 (3.11–3.68)	<0.001	1.95 (1.70–2.21)	<0.001	1.91 (1.70–2.12)	<0.001
60–69 years	5.83 (5.19–6.48)	<0.001	7.84 (7.09–8.60)	<0.001	2.33 (2.01–2.66)	<0.001	2.28 (2.00–2.27)	<0.001
Social class I	1		1		1		1	
Social class II	1.15 (1.12–1.19)	<0.001	1.19 (1.14–1.25)	<0.001	1.20 (1.16–1.25)	<0.001	1.18 (1.14–1.22)	<0.001
Social class III	1.35 (1.28–1.43)	<0.001	1.39 (1.26–1.52)	<0.001	1.48 (1.36–1.60)	<0.001	1.49 (1.39–1.59)	<0.001
Non-smokers	1		1		1		1	
Smokers	1.22 (1.17–1.27)	<0.001	1.12 (1.08–1.16)	<0.001	1.57 (1.50–1.64)	<0.001	3.72 (3.46–3.99)	<0.001
Adherence to the Mediterranean diet	1		1		1		1	
Non-adherence to the Mediterranean diet	1.64 (1.49–1.80)	<0.001	2.14 (2.00–2.28)	<0.001	4.98 (4.59–5.39)	<0.001	4.50 (4.02–4.99)	<0.001
Perform physical activity	1		1		1		1	
Physical inactivity	8.54 (7.73–9.35)	<0.001	3.11 (2.90–3.33)	<0.001	7.62 (7.30–7.93)	<0.001	8.61 (7.70–9.61)	<0.001
SF-12 good	1		1		1		1	
SF-12 poor	2.24 (2.15–2.33)	<0.001	1.59 (1.43–1.76)	<0.001	4.28 (3.97–4.58)	<0.001	3.66 (3.25–4.07)	<0.001

TC, Total cholesterol. HDL, High density lipoprotein. LDL, Low density lipoprotein. TG, Triglycerides. SF-12, Short Form Health Survey. OR, Odds ratio. CI, Confidence interval.

## Data Availability

The data presented in this study are available on request from the corresponding author due to confidentiality obligations.

## Supplementary Materials

The following supporting information can be downloaded at: https://www.mdpi.com/article/10.3390/jcm14196876/s1, STROBE Checklist–Cross-Sectional Studies.
