# Supplementary material for: Sociodemographic, Lifestyle, and Quality of Life Determinants of Atherogenic Risk: A Cross-Sectional Study in a Large Cohort of Spanish Workers
Source: J Clin Med. 2025 Sep 28;14(19):6876. doi: 10.3390/jcm14196876 (PMC12524400; doi:10.3390/jcm14196876)
Supplement: Supplementary file 1 [file jcm-14-06876-s001.zip › jcm-3839842-supplementary.pdf]

STROBE Checklist – Cross-Sectional Studies

Section	Item	Recommendation	Check
**Title and Abstract**	1	Indicate the study's design with a commonly used term (e.g., "cross-sectional") in the title or abstract.	X
**Introduction**	2	Explain the scientific background and rationale for the investigation.	X
	3	State specific objectives, including any pre-specified hypotheses.	X
**Methods**	4	Present key elements of study design early in the paper.	X
	5	Describe the setting, locations, and relevant dates, including recruitment, exposure, follow-up, and data collection periods.	X
	6	Give eligibility criteria, sources and methods of participant selection, and methods of sampling.	X
	7	Clearly define all outcomes, exposures, predictors, potential confounders, and effect modifiers.	X
	8	For each variable, give sources of data and details of methods of assessment. State if assessment methods were comparable across groups.	X
	9	Describe any efforts to address potential sources of bias.	X
	10	Explain how the study size was arrived at.	X
	11	Explain how quantitative variables were handled in the analyses. If applicable, describe which groupings were chosen and why.	X
	12	Describe all statistical methods, including those used to control for confounding.	X
	12a	Describe methods used to examine subgroups and interactions.	X
	12b	Explain how missing data were addressed.	X

Section	Item	Recommendation	Check
	12c	If applicable, describe analytical methods taking account of sampling strategy.	X
	12d	Describe any sensitivity analyses.	X
**Results**	13	Report the number of individuals at each stage of the study (e.g., potentially eligible, examined for eligibility, confirmed eligible, included in the study, analysed). Consider a flow diagram.	X
	14	Give characteristics of study participants (e.g., demographic, clinical, social) and information on exposures and potential confounders.	X
	14a	Indicate the number of participants with missing data for each variable of interest.	X
	15	Report outcome data and main results with estimates of precision (e.g., 95% confidence intervals). Clearly state which confounders were adjusted for and why.	X
	15a	If relevant, report category boundaries when continuous variables were categorized.	X
	16	Report other analyses done (e.g., analyses of subgroups, interactions, sensitivity analyses).	X
**Discussion**	17	Summarize key results with reference to study objectives.	X
	18	Discuss limitations of the study, considering potential sources of bias or imprecision. Discuss both direction and magnitude of any potential bias.	X
	19	Provide a cautious overall interpretation of results considering objectives, limitations, multiplicity of analyses, results from similar studies, and other relevant evidence.	X
	20	Discuss the generalizability (external validity) of the study results.	X
**Other Information**	21	Give the source of funding and the role of funders.	X

Section	Item	Recommendation	Check
22 (if applicable)	Indicate if the study protocol is available or registered.	Not applicable	
